# Shared Disease Mechanisms in Neurodevelopmental Disorders: A Cellular and Molecular Biology Perspective

**DOI:** 10.3390/brainsci16010054

**Published:** 2025-12-30

**Authors:** Elizabeth A. Pattie, Philip H. Iffland

**Affiliations:** Department of Neurology, University of Maryland School of Medicine, Baltimore, MD 21201, USA; epattie@som.umaryland.edu

**Keywords:** cortical malformations, epilepsy, seizures, intellectual disability, developmental delay, brain development

## Abstract

Neurodevelopmental disorders (NDDs) are defined as a group of conditions that result from impaired brain development. Disorders that are commonly classified under NDDs include intellectual disability (ID), autism spectrum disorder (ASD), attention-deficit/hyperactivity disorder (ADHD), communication and learning disorders, developmental delay (DD), brain malformations, cerebral palsy, Down syndrome, schizophrenia, and childhood epilepsies. A significant hinderance in the development of targeted treatments for NDDs are gaps in understanding how underlying genetic changes alter cellular physiology and how these changes may converge or diverge across NDDs with similar symptoms. Here, we focus on the genetic overlap between epilepsy, ASD, and other NDDs to identify common cellular and molecular mechanisms that may inform future treatments for each of these disorders individually or together. We describe several genes—including *CDKL5, TSC1/2, SCN1a,* and *TANC2*—that have been associated with epilepsy, ASD, or other NDD phenotypes that play a critical role in regulating one or more stages of brain development or function but differ widely in their disease-causing mechanisms. We also describe genotype–phenotype relationships. Finally, how a gene may cause NDDs through distinct functional pathways, or where different types of pathogenic variants within the same gene can have significantly different phenotypic outcomes is detailed.

## 1. Introduction

Although diagnostic criteria and classifications can vary, neurodevelopmental disorders (NDDs) are defined as a group of conditions that result from impaired brain development [1,2,3,4]. Disorders that are commonly classified under NDDs include intellectual disability (ID), autism spectrum disorder (ASD), attention-deficit/hyperactivity disorder (ADHD), communication and learning disorders including developmental delays (DDs), as well as tic and motor disorders [1,2,3,4]. Additionally, disorders such as brain malformations, cerebral palsy, hearing and vision loss, Down syndrome, schizophrenia, and childhood epilepsies are grouped in or associated with NDDs [1,2,3,4]. These conditions typically present early in life and can be challenging to diagnose due to their complex and heterogenous array of phenotypes [1,2,3,4,5].

Epilepsy is a particularly challenging component of many NDDs. Children with NDDs and epilepsy are at a higher risk of poor long-term health outcomes, especially as it pertains to neural dysfunction, DD, and disability [1,3]. While not all NDDs are genetically driven, epilepsy associated with NDDs that are caused by genetic variants are more likely to be refractory, have earlier seizure onset, and worse overall developmental outcomes. Thus, understanding the prevalence of epilepsy and NDD comorbidities can provide meaningful insights into shared etiologies. Further, the global prevalence of epilepsy and ASD is approximately 52 million and 62 million people, respectively [6,7]. Compared to a 1–2% prevalence in the general population, early childhood comorbidity of epilepsy co-occurring with ASD, and vice versa, has a prevalence of 11% and 8%, respectively [8]. Although intellectual disability and epilepsy were not the most prevalent disorders within this demographic, they were associated with the largest non-fatal burden of disease [3]. Overall, the prevalence and global burden of epilepsy and neurodevelopmental disorders remain relatively unchanged since 1990 despite our increased understanding of the genetic and mechanistic underpinnings of these complex disorders [3].

Another particularly challenging subset of NDDs are developmental and epileptic encephalopathies (DEEs). DEEs are early-onset genetic epilepsies characterized by developmental and cognitive delays, accompanied and worsened by frequent seizures that are often medically refractory [8,9,10,11,12]. Patients with DEEs are at a higher risk of status epilepticus and developmental regression, and even when seizure control can be achieved, developmental and cognitive deficits persist [10]. Early detection and medical intervention can significantly improve long-term outcomes, but treatment efficacy is highly dependent on the underlying genetic etiology [13,14,15,16].

A significant hinderance in the development of targeted treatments for DEEs and NDDs is that much is still unknown about the etiologies of genetic epilepsy, NDDs, and DEEs, and to what degree the underlying mechanisms of these disorders converge or diverge. However, there is a high association, in both genetic epilepsies and NDDs, between the developmental timeline of gene expression, age of symptom onset, and co-occurrence of overlapping phenotypes. For example, a recent study analyzed nearly 240 ASD-related traits across over 5000 individuals and was able to classify these individuals into four distinct phenotypically defined groups [17]. Two of the groups, classified as the “mixed ASD with DD” group and the “broadly affected” group, had similar levels of developmental delay traits, including cognitive and language impairments, as well as significantly younger age of diagnosis and larger degree of delayed developmental milestones compared to the other two groups. Genotypically, all groups were enriched for de novo variants across known ASD-related genes, although prevalence was highest in the broadly affected group, while the “mixed ASD with DD” group had significant prevalence of both high-impact de novo and rare inherited variants. Lastly, they also found that the “mixed ASD with DD” group was enriched for genes that are highly expressed during fetal and neonatal development with declined expression during later stages, and the broadly affected group showed dysregulation across all developmental stages. Thus, while the association between epilepsy, ASD, and NDDs is well known, understanding how and when disease mechanisms converge may yield novel therapeutic targets and/or approaches.

Concerted efforts to define the relationship between genetic variants and clinical phenotypes have resulted in several curated databases of disease-associated genes. For example, SFARI contains over 900 genes with high confidence or those that are considered strong candidates to be associated with ASD. Further, OMIM lists over 120 genes associated with DEE. The Geisinger Developmental Brain Disorder Gene Database contains over 200 genes to be high-confidence candidates for pathogenic LoF variants. Furthermore, SysNDD contains nearly 2000 genes categorized as having a definitive relationship with DD, ID, or ASD. In this review, we focus on the genetic overlap between epilepsy, ASD, and other NDDs to identify common mechanisms that may inform future treatments for each of these disorders. Herein we focus specifically on the most common causes of overlapping ASD, ID, and epilepsy. In addition, we have focused on genes that impact brain development that are often overlooked. Indeed, ion channel and synaptic protein variants can have a significant impact on the developing brain. Thus, we describe several genes that have been associated with epilepsy, ASD, and other NDDs that play a critical role in regulating one or more stages of embryonic development and brain formation but differ widely in their mechanisms (Table 1). We also describe genotype–phenotype relationships and how a gene may cause epilepsy and NDDs through distinct functional pathways, or where different types of pathogenic variants within the same gene can have significantly different phenotypic outcomes. Although the information described here, regarding genes linked to NDDs, is not exhaustive, we describe in detail the breadth and complexity of mechanisms that orchestrate brain development and the common areas in which brain development is disrupted in these disorders. Details of each gene discussed are based on the relevance to this review and/or the depth of the literature available on that specific gene.

## 2. Ion Channel Dysregulation

The first-line approach to treating epilepsy is with antiseizure medications (ASMs). The primary mode of action for ASMs involves modulating neurotransmitter release and activation/inhibition of synaptic receptors or ion channels to prevent abnormal action potential firing [18]. Indeed, many epilepsy-causing genetic variants of ion channel-encoding genes have been identified. Considering the fundamental role of synapses in brain function, and that postnatal development is a critical period of synapse formation and network connectivity of maturing neurons, many epilepsy-causing ion channel genes are also unsurprisingly associated with NDD comorbidity or DEEs. Furthermore, many ion channels are also highly expressed across a range of cell types during embryonic and neural development, and can contribute to a variety of cellular processes, such as proliferation, migration, and differentiation, throughout neurodevelopment [19,20]. Indeed, genetic variants in ion channels do not simply alter electrophysiology that results in abnormal network activity. Ion channel variants can alter the way the brain develops—setting up abnormal networks—and impact their function once networks are established.

### 2.1. Voltage-Gated Calcium Channels

Many stages of embryonic and postnatal neurodevelopment are dependent on calcium, such as proliferation of radial glial cells, migration of excitatory and inhibitory neurons, apoptosis, differentiation, synaptogenesis, maturation, and plasticity [19,21]. For example, ion channel activity is crucial for regulating internal Ca^2+^ concentration gradients, and the assembly and disassembly of actin is highly dependent on these gradients [22]. Thereby, ion channel-dependent Ca^2+^ gradients facilitate cytoskeleton morphology and cell polarity changes via modulating actin dynamics that are necessary for a variety of processes, including proliferation, cell migration, and growth cone guidance [22,23]. Ion channel-related deficits in prenatal neuronal migration can cause epileptogenic malformations of cortical development that are also frequently associated with ASD [20].

Voltage-gated calcium channels are one of the primary regulators of neuronal Ca^2+^ influx [21]. Genetic variants in several voltage-gated calcium channel genes are associated with NDDs or epilepsy, with *CACNA1A*, *CACNA1C*, and *CACNA1E* being some of the most commonly affected [24]. *CACNA1C* is associated with syndromic ASD, but also causes a cardiovascular disorder, Timothy syndrome, which can cause seizures or sudden death due to cardiac arrhythmias [25]. Variants in *CACNA1A* are associated with syndromic ASD, DEE, DD, severe ID, hypotonia, and ataxia [26,27,28]. Furthermore, *CACNA1A*-related DEE is characterized by early infantile onset, with refractory seizures beginning within the first hours to days of life [26,27,28]. For *CACNA1E*, DEE is associated with early-onset refractory seizures, ASD, profoundly impaired development, but is also associated with macrocephaly [29].

### 2.2. Voltage-Gated Sodium Channels

Some of the most frequent forms of genetic epilepsies and NDDs are due to variants in the voltage-gated sodium channel genes such as *SCN1A, SCN2A, SCN3A,* and *SCN8A* [30]. *SCN1A* is one of the most prevalent epilepsy-causing genes and causes Dravet syndrome—a severe form of DEE [31]. Additionally, *SCN1A* is associated with the onset of refractory epilepsy and status epilepticus (despite normal development prior to onset) within the first year of life, leading to motor impairment, cognitive decline, syndromic ASD, and ID [31]. For *SCN3A,* genetic variants are associated with DEE, profound ID, polymicrogyria, and onset of medically refractory seizures within the first few weeks of life [30,32]. This earlier age of onset, as compared to *SCN1A,* is due to their distinct expression patterns [30,32]. *SCN1A* expression is most abundant in postnatal interneurons, while *SCN3A* is predominantly expressed during embryonic and neonatal stages of brain development and decreases with age [32,33]. Furthermore, *SCN3A* is highly expressed in the cortical plate, including outer radial glia [32]. This cell type is crucial for the formation of gyri during brain development, and this is likely responsible for the association between *SCN3A* and polymicrogyria [32].

*SCN2A* and *SCN8A* are closely related genes that are both expressed in excitatory neurons [30,34]. With *SCN2A*, severe and refractory seizures begin during neonatal or infantile stages and often result in spontaneous or treatment-responsive seizure remission later in childhood [35]. In addition to epilepsy and DEE, *SCN2A* is one of the most prevalent genetic causes of ASD and is also associated with complex NDD, DD, severe ID, ADHD, episodic ataxia, hypotonia, microcephaly, and white matter defects [35]. For *SCN8A*, genetic variants are associated with medically refractory epilepsy that often begins within the first year of life, as well as DEE, DD, ID, developmental regression, hypotonia, and ataxia [36]. While *SCN2A* expression begins prenatally and remains high across the lifespan, *SCN8A* expression increases with age, similarly to *SCN1A* [30]. There is a functionally compensatory relationship between *SCN2A* and *SCN8A*, which may contribute to the spontaneous nature of seizure remission that can occur with *SCN2A*, due to this delayed expression of *SCN8A*, and provides a target for therapeutic interventions, such as PRAX-562, a sodium current inhibitor currently undergoing clinical trials for both *SCN2A*- and *SCN8A*-DEE (EMBOLD study, NCT05818553) [30,37].

### 2.3. Potassium Channels

Potassium (K^+^) channels are a highly diverse group of ion channels that are associated with both epilepsy and ASD. Several of them play important roles during neurodevelopment, including stem cell and neural progenitor proliferation, migration, and neuronal differentiation [38]. For instance, *KCNA1* and *KCNA5* (Kv1.1 and Kv1.5) channel-mediated hyperpolarization is critical for regulating intracellular Ca^2+^ levels during wound healing, and *KCNA3* (Kv1.3) channels modulate membrane potential, activity-dependent signal transduction, and cell cycle progression [22,39]. *KCNA2* is associated with normal early development, followed by seizure onset between 5 and 17 months of age, leading to DD, developmental regression, and ID. Interestingly, despite *KCNA2*-related seizures often being initially refractory, seizure remission can occur later in childhood [40,41,42]. However, in those cases, cognitive deficits persist.

Loss-of-function (LoF) variants in *KCNB1* (Kv2.1) are associated with DEE, onset of seizures, and developmental delays in late infancy, and about half of patients will also have autistic features or behavioral abnormalities [43,44]. During embryonic development, Kv2.1 regulates cortical neuron migration by interacting with integrin to activate focal adhesion kinase (FAK) to promote cell adhesion, polarization, and migration [20,22,45,46,47].

Expression of *KCNC1*, which encodes the delayed rectifier K^+^ channel subtype Kv3.1, begins during early stages of embryonic development and increases across neurogenesis and neuronal maturation [38,48,49,50]. Furthermore, Kv3.1 promotes cell proliferation and differentiation for embryonic and adult neural precursor cells (NPCs) and neuroblasts [38,48,49,50]. Genetic variants in *KCNC1* can cause progressive myoclonus epilepsy, and in some cases ID/DD and DEE [38,48,49,50].

While there are clear roles for these channel subtypes in cell migration and proliferation, the differences in disease phenotypes across channels may not only represent functional differences but also cell type-specific and temporal differences in expression during and after brain development.

#### 2.3.1. Two-Pore Potassium Channels

During brain development, two-pore domain K^+^ channels (K2P or KCNK) undergo a significant degree of differential spatiotemporal expression patterns [51,52]. For example, *KCNK3* (encoding TASK-1) expression begins in neonatal post-migratory neurons, while *KCNK9* (encoding TASK-3) is highly expressed in proliferating cells during embryonic brain development and regulates activity-dependent cortical migration [52,53]. Birk–Barel, or *KCNK9*-Imprinting, syndrome is an NDD that causes heterogeneous features including speech and motor delay, ID, behavioral abnormalities, and knockdown (KD) of *KCNK9*; *KCNK2* and *KCNK10* (encoding TREK-1 and TREK-2, respectively) also cause impaired morphological maturation and cortical heterotopia of late-born excitatory neurons [53,54]. For *KCNK2* in particular, TREK-1 has been shown to colocalize with and induce the remodeling of actin cytoskeleton in fetal neurons, contributing to the formation of both dendritic and axonal filopodia and growth cones, playing an important role in axon migration and guidance as well as synaptogenesis [55]. The role of TREK-1 on the central nervous system (CNS) also extends into adulthood, including both neurodegeneration and neuroinflammation [56,57,58]. Genetic variants in TREK1 can cause cortical atrophy and downregulation in blood–brain barrier (BBB) endothelial cells in response to inflammatory cytokines such as interferon-γ (IFN-γ) and tumor necrosis factor-α (TNF-α) [57,58].

#### 2.3.2. Kv7 Voltage-Gated Potassium Channels

In adult neurons, Kv7 channels localized to the axon initial segment (AIS) and nodes of Ranvier, play a role in setting membrane potentials and activation thresholds, and, in particular, *KCNQ2* and *KCNQ3* (encoding Kv7.2 and Kv7.3) are highly associated with neuronal excitability and seizure susceptibility [12,48,59,60,61].

Pathogenic variants of *KCNQ2* can have either LoF, GoF, or dominant negative LoF effects, each associated with distinct epilepsy phenotypes, including benign familial neonatal epilepsy, infantile spasms without neonatal seizures, or neonatal DEE, respectively [12,62,63,64,65,66,67]. In fact, the most common genetic cause of neonatal-onset DEE is variants in *KCNQ2*, where seizures begin within the first week of life, and the majority of patients will maintain moderate to severe ID/DD despite seizure cessation [12,62,63,64,65,66,67,68]. Some *KCNQ2* variants have also been linked to ASD and autistic features [69,70,71].

To further highlight the critical but complex role that *KCNQ2* plays in the CNS, *KCNQ2* transcripts undergo alternative splicing with differential exon expression from stem cells to NPCs to differentiated neurons, and although the degree to which the distinct products of these transcripts functionally differ remains unclear, the developmental transcript does lack measurable K^+^ current activity [12,66,67,72]. Based on temporal expression patterns, *KCNQ2* is believed to promote cell cycle progression during early developmental stages, eventually shifting to roles that promote neuronal differentiation and synaptogenesis [12,48,66,67,72]. Furthermore, inhibition or knockdown (KD) of *KCNQ2* during in vitro NGF-induced differentiation promotes neurite outgrowth, whereas activation or overexpression had the inverse effect [73]. While the exact mechanisms by which variants in *KCNQ2* result in NDDs are unknown, it is likely that the changes in cell cycle early in development alter the number, type, and location of neurons and, at later time points, impairment in network function results in persistent behavioral changes and seizures.

#### 2.3.3. Sodium-Activated Potassium Channels

*KCNT1* and *KCNT2* encode for sodium-gated potassium channel subunits Slack (KNa1.1) and Slick (KNa1.2), respectively, which are structurally similar but have distinct kinetic properties and expression patterns [74,75,76,77,78,79]. Both are highly expressed in the brain, although in the adult rat CNS, *KCNT2* expression is greater than *KCNT1* within the hippocampus as well as layers II, III, and V of the neocortex [78,79,80,81]. During embryonic corticogenesis, *KCNT2* expression is initially localized to the developing subplate and marginal zones but becomes more ubiquitous within the cortex during development [79,81].

Pathogenic *KCNT1* variants are almost always gain-of-function (GoF) and cause severe early-onset epileptic disorders with refractory seizures and are associated with ID, psychiatric features, and arrest of psychomotor development following seizure onset, while *KCNT2*-related DEE can be caused by change-of-function, GoF, or LoF variants but is also associated with ID and developmental deficits [61,74,75,80,82,83,84,85].

Functionally, the activity of Slick and Slack are differentially regulated. Slick is more rapidly activated than Slack and has higher sensitivity to changes in Cl^−^ and Na^+^ levels [78]. Both are activated by high levels of intracellular NAD^+^, but Slick also has a nucleotide-binding site that causes channel inhibition upon ATP binding, while Slack has an intercellular binding domain that interacts with FMRP for reciprocal regulation [74,76,78,80,81,86,87,88]. For Slick, these regulatory mechanisms are likely important for neuroprotection by maintaining energy homeostasis and promoting cell viability. In response to hypoxia, epileptiform activity, or high frequency of action potential firing, activation of Slick by elevated NAD^+^ or by loss of ATP inhibition results in hyperpolarization and reduced excitability [78,80,81]. This suggests that Slick plays a role in conserving energy consumption by decreasing neuronal excitability when the ATP levels are depleted, thereby preserving energy homeostasis. Considering the spatiotemporal expression of *KCNT2* during neurodevelopment and corticogenesis, as well as in the adult CNS, Slick may play a physiological role in protecting cell viability throughout neuronal differentiation and maturation, especially during synaptogenesis and network connectivity. LoF variants in *KCNT2* would have a clear impact on neuronal maturation and function. However, how GoF variants would result in similar challenges is unclear.

The intracellular domain of Slack has an FMRP binding domain rather than ATP. FMRP is a translation-suppressing RNA-binding protein that is broadly expressed in mature neurons and glial cells, as well as neural progenitors during development, and loss of FMRP is the most common monogenetic cause of inherited ID and ASD, which can be accompanied by seizures [76,87,88,89]. FMRP localizes to dendrites, in association with ribosomal complexes, to regulate activity-dependent translation of proteins involved with synaptic plasticity. Interestingly, FMRP also plays a broader role in neurodevelopment, from neural progenitor proliferation, cell fate specification and migration, axonal outgrowth, dendrite morphology, as well as synapse formation [88]. Whether or not the interaction between Slack and FMRP is involved in broad neurodevelopmental processes, it does facilitate the localization and activity of FMRP in dendrites as well as increasing Slack activity, and there is clear evidence that dendritic dysfunction can result in NDD, and particularly ASD, phenotypes [76,86,87,88].

#### 2.3.4. HCN Channels

Hyperpolarization-activated cyclic nucleotide-gated channel genes (*HCN1*, *HCN2*, *HCN3*, and *HCN4*) are crucial for regulating electrical activity in heart cells as well as neurons. While their roles in neurodevelopment and cardiovascular phenotypes are variable, variants in these channels are associated with epilepsy, neuropsychiatric disorders, and NDDs including ASD, ID, and DD. HCN channels are expressed throughout the brain and primarily function as a “pacemakers” to modulate action potential frequency by regulating Na^+^ and K^+^ flux to induce inward currents in response to membrane hyperpolarization [90,91,92]. Due to sequence homology across HCN channels, and their role in both epilepsy and cardiovascular function, development of precision medicine to target variant-specific epileptogenic mechanism has been challenging [91]. However, it is worth noting that although the pathogenesis remains unclear, there may be a link between cardiorespiratory function and sudden unexplained/unexpected death in patients with epilepsy (SUDEP) [93,94].

HCN channels are not just involved in regulating the activity of established neural networks, as expression of these channels begins early during development, although levels vary by gene, developmental stages, and brain regions [91,95,96]. In embryonic stem cells, HCN inhibition significantly impairs cell cycle progression but not cell viability or differentiation state—which causes microcephaly in mice [96,97,98]. During later stages of neurodevelopment, these channels act as “molecular switches” in promoting the maturation of differentiating neurons during the beginning stages of synaptogenesis by inhibiting short-term depression, specifically in immature neurons in order to strengthen newly formed network connections [95,99]. Reciprocally, network activity also regulates HCN channel expression and activity, and this feedback is necessary to fine-tune network maturation [95,99]. Therefore, disturbances in this process, either by environmental influence (e.g., hypoxia, nicotine exposure, and alcohol exposure) or functional abnormalities in HCN channel activity, can significantly alter how neural networks develop and cause long-lasting CNS dysregulation [95,100,101,102,103].

Of the HCN channels, *HCN1* has a particularly low tolerance for genetic variation, the most well-defined association with epilepsy, and can cause generalized epilepsy or more severe DEE characterized by infantile onset of refractory seizures, severe ID/DD, atypical Rett syndrome, microcephaly, movement dysfunction, and behavioral abnormalities [91,92,96,104,105,106]. Interestingly, the phenotypic outcomes of pathogenic variants are closely linked to the variant type. More specifically, missense variants (regardless of LoF, GoF, or dominant negative effect) are more associated with seizure phenotypes, whereas nonsense variants are more likely to cause ID and DD [91], though the exact mechanism for these differences is poorly defined.

*HCN2* is a major contributor to the role that HCN channels play throughout embryonic development and is involved in neural plate and CNS patterning, proliferation and migration of stem cell and neural progenitors, biomechanical modulation of signal transduction pathways, and oligodendrocyte myelin formation [100,101,102,103,107]. For *HCN2*, GoF variants are mostly associated with epilepsy, but LoF variants are associated with moderate to severe ID/DD, in addition to epilepsy or DEE [91,92,108].

Little is known about the role of *HCN3* and *HCN4* in brain development. *HCN3* is tolerant to genetic variants, although pathogenic variants associated with epilepsy have recently been discovered in individuals that responded well to treatment [109]. *HCN4* pathogenic variants are most strongly linked to cardiovascular disease but can also be associated with generalized epilepsy and lasting cognitive impairments [110,111]. Studies in *Xenopus* embryos have demonstrated that *HCN4* expression begins during very early stages of development, where it plays a particularly important role in establishing left-right axis, in a manner that is independent of canonical Nodal-Lefty asymmetric patterning mechanisms, possibly by altering regulation of metabolic physiology [112]. Improper establishment of left-right patterning is associated with birth defects, especially of the heart and brain [112]. Although important during embryonic development, *HCN4* expression levels reduce over time to the point where there is generally little to no expressed in neurons after birth, instead being replaced by *HCN1* [113,114]. Interestingly, in both mouse models and surgically resected human brain tissue of tuberous sclerosis complex (TSC) and focal cortical dysplasia type II (FCDII), dysmorphic cortical neurons show elevated *HCN4* that was dependent on mTOR pathway hyperactivation, thereby pathogenically linking *HCN4* to malformations of cortical development (MCDs) and further establishing the link between immature neuronal phenotypes and neurons within malformed cortex [113].

## 3. Cell Signaling Cascades and Other Molecular Pathway Dysfunction

Malformations of cortical development (MCDs) are a broad group of disorders characterized by abnormal formation of the neocortex, and are a common cause of childhood epilepsy, ID, DD, ASD, or other NDDs, with a wide range of variation in severity and comorbidity depending on the etiology [115,116,117]. Medically refractory epilepsies are common in MCD subtypes [118]. Genetic causes of MCD can be due to germline or somatic variants [118]. Detection of somatic variants is particularly challenging in MCD and requires access to brain tissue specimens. However, improvements in high-resolution MRI have enhanced our ability to detect MCD [118]. As more MCD genes have been discovered, it has become clear that epilepsy is not just a disorder of dysregulated synapses and ion channels, but that dysregulation of brain development can impart lasting phenotypic consequences even when ion channels are normally functioning [116,117]. Initial classification methods aiming to categorize MCDs have focused on the developmental stage of disease onset, in particular cellular proliferation, neuronal migration, and cortical organization [115,116,119]. For example, microcephaly and macrocephaly are both generally associated with dysregulation of cell cycle progression or cell viability as a result of altered quantity of cells in the brain [116].

Our understanding of embryonic brain development and genetics has vastly improved since these first classifications were developed, and it is clear that these three stages of development are not temporally distinct. Furthermore, many of these genes have multiple biological functions, making categorization of these disorders challenging, and have since shifted towards classifying by genetic and molecular etiology [115]. Several structural abnormalities associated with MCD have been described, generally detectable by neuroimaging, including microcephaly, megalencephaly, lissencephaly, cobblestone malformation, heterotopias, focal cortical dysplasia, tuberous sclerosis complex, polymicrogyria, and many others [120,121,122]. Several molecular pathways have also been linked to MCD, encompassing many complex and interconnecting mechanisms that regulate embryonic and neuronal development. The key steps involved in these processes are (1) rapid cellular proliferation, (2) migration of newly formed cells into discrete regions, and (3) differentiation of these cells to form functional organ systems. These processes involve coordinated regulation of cell physiology including nutrient sensing and consumption, energy expenditure, signal transduction pathways, transcriptional programs, subcellular organization, and cytoskeletal dynamics.

Rather than systematically reviewing all MCD genes, here we focus on several genes associated with MCD, epilepsy, and ASD to illustrate the broad range of etiological mechanisms and cellular pathways that shape brain development as well as the overlapping cellular pathologies that result in epilepsy, ASD, and NDD within the same gene.

### 3.1. ANKRD17

Ankyrin repeat domain 17 (*ANKRD17*) variants are associated with syndromic complex NDDs and haploinsufficient-sensitive syndromic ID [123,124]. In particular, it causes Chopra–Amiel–Gordon syndrome, which is characterized by a variable degree of DD, ID, and speech delay, but can also be accompanied by additional features, including non-specific brain abnormalities, other neurodevelopmental disorders such as ASD and ADHD, feeding difficulties, growth failure, abnormal EEG and/or epilepsy, joint hypermobility, gait/balance disturbances, and recurrent infections [123,124].

The encoded protein ANKRD17, also known as GTAR or MASK2, is named based on the presence of ankyrin repeats—a structural motif present in a range of proteins that are associated with various protein–protein interactions that can play a role in regulating signal transduction, cytoskeletal organization, and transcription [123,125]. Along with the paralog ANKHD1 (MASK1), ANKRD17 was first identified as the mammalian homologs to the Mask protein discovered in *Drosophila* [123,126,127,128]. In *Drosophila*, Mask binds to Yorkie (Yki), the sole fly homolog to mammalian yes-associated protein (YAP, also known as YAP1) and transcriptional coactivator with PDZ-binding motif (TAZ, also known as WWTR1) [123,126,127,128]. This interaction downregulates the tumor-suppressing activity of the Hippo signaling pathway by facilitating the nuclear transport and transcriptional coactivation levels of Yki, to positively regulate cell cycle progression [123,126,127,128,129].

In mammalian cells, both MASK1 and ANKRD17 regulate YAP activity in a manner comparable to Mask and Yki. Overexpression of either *MASK1* or *ANKRD17* increases nuclear YAP localization and activity while siRNA-induced KD had an inverse effect, and double siRNA KD of both *MASK1* and *ANKRD17* induces in vitro apoptosis [128,130]. Further, Ankrd17 is critical for regulating several cellular pathways, especially proliferation and survival. Upon phosphorylation by Cyclin E/Cdk2, Ankrd17 interacts with DNA replication factors CdC6 and PCNA to promote the initiation of S-phase [131]. Furthermore, in addition to inhibiting DNA replication and cell proliferation, KD of *Ankrd17* also upregulates the expression of both *p53* and *p21* [131]. In human liver cells, overexpression of *ANKDR17* increases the activity of YAP1, AKT, DDR1, and STAT3, leading to dysregulation of several key signaling pathways, ultimately having an oncogenic effect by increasing cell proliferation, survival, migration, and pro-metastatic invasion capability, while KD has an inverse effect [130]. Interestingly, in addition to regulating STAT3, Ankrd17 also has a pro-inflammatory role by regulating Nod1, Nod2, and RIG-I-like receptor pathways [132,133], which may explain why patients have an increase susceptibility to recurrent infections.

### 3.2. PHACTR1

Phosphatase and actin regulator 1 (*PHACTR1*) variants are associated with DEE and West syndrome [134]. It is one of several genes associated with West syndrome which is characterized by infantile spasms, hypsarrhythmia, developmental arrest or regression, ID, ASD, and cerebral atrophy or dysgenesis such as lissencephaly and hydrocephalus [134]. Beyond neurodevelopment, *PHACTR1* is also associated with fibromuscular dysplasia, myocardial infraction, and coronary artery disease [134,135].

*PHACTR1*, also known as RPEL repeat-containing 1, encodes for one of the four members of the PHACTR family of protein phosphatase 1 (PP1)-binding proteins [136,137,138]. These proteins contain G-actin binding motifs and modulate PP1 activity and actin cytoskeletal structure; however, they each have unique expression patterns by cell type and developmental stages [134,136,137,139]. Expression of *PHACTR1* is restricted to the brain, especially in neurons of the cortex, hippocampus, and striatum [134,136,139]. *Phactr1* also has differential gene expression and protein localization [139]. In mice, *Phactr1* expression is high during embryonic stages of cortical development, especially in the nucleus of cells located in cortical plate as well as ventricular and subventricular zones [134,137,139]. This nuclear localization is unique to Phactr1 as compared to other members of the Phactr family [137]. Expression of *Phactr1* is lowest postnatally but has a more distributed localization between the nucleus and cytoplasm of cortical neurons of the lower cortical plate and the intermediate zone [134,137,139]. Expression then increases again until prepubertal stage, with more diffuse subcellular localization, including synapses [134,136,137,139].

Altered cytoskeletal dynamics associated with loss of *PHACTR1*, dysregulation of PHACTR1/PP1 activity, or impaired G-actin interaction leads to defects of cell motility and morphology [134,138,140,141,142,143,144]. For example, disrupted regulation of actin polymerization due to Phactr1 depletion causes impaired lamellipodial dynamics [144]. In cancer cells, PHACTR1 enhances cell motility and invasiveness [137,140,143]. Ultimately, PHACTR1 regulates actin dynamics, playing a key role in regulating migration and arborization of neurons during development; furthermore, it may contribute to synapse formation and plasticity. Impairment of these processes ultimately results in abnormal neuronal positioning within the brain as well as abnormal neuronal connectivity and network function.

### 3.3. CNTNAP2

Variants in contactin-associated protein 2 (*CNTNAP2*) have a strong association with ASD, which is also the cause of syndromic NDDs including Pitt–Hopkins-like syndrome 1 and cortical dysplasia–focal epilepsy (CDFE) syndrome [145,146,147,148,149,150]. Autosomal recessive CDFE was first identified in Old Order Amish children due to a homozygous LoF of *CNTNAP2* and is characterized by early-onset of refractory focal seizures, mild macrocephaly and cortical malformations, ID/DD with regression following seizure onset, autistic traits, ADHD, as well as impulsive and aggressive behaviors [145]. Similar phenotypes have been reported in Pitt–Hopkins-like syndrome 1 [151,152]. Focal and unilateral dysplasia in the cortex was detected in some individuals by neuroimaging, and histological analysis of brain resections also demonstrated several abnormalities suggestive of impaired cortical development [145]. Interestingly, surgical intervention is unsuccessful at maintaining long-term seizure control [145].

The encoded protein of *CNTNAP2*, CASPR2, is a neuronal transmembrane protein of the neurexin family, and can be found along myelinated axons, localized to juxtaparanodes where it binds to CNTN2/TAG1 in glial cells to facilitate cell–cell adhesion [145,146,153,154]. CASPR2 also binds with other cell-adhesion partners, including CNTN1, DLG1, and DLG4 [155]. However, embryonic expression of *CNTNAP2* suggests that *CNTNAP2* also plays a role in neurodevelopment [146,156,157,158]. This is supported by the finding that the Caspr2 myelin-related binding partner, Tag-1, also has embryonic expression, where it plays a role in regulating neuronal migration [146]. Furthermore, single cell RNA-sequencing and proteometabolomic studies of *Cntnap2* KO mice revealed that in addition to altered networks related to axons and myelin, *Cntnap2*-deficiency also caused dysregulation of pathways involved in mitochondrial function, synapse activity and vesicle transport, and neuronal projections [159].

Caspr2 has recently been shown to undergo sequential secretase-dependent proteolytic cleavage, and the resulting domain fragments of this posttranslational modification have unique functional properties that are critical for development [156,157,160]. The extracellular domain that is released can bind and enhance the activity of the calcium pump PMCA2 [160]. The CASPR2 intracellular domain (CICD) contains a PDZ domain that has been shown to bind to and facilitate recruitment of CASK to the plasma membrane while CASPR2 remains intact as a transmembrane protein, whereas proteolytic release of CICD enhances the transcription factor activity of CASK via nuclear translocation [156,161]. Additionally, the PDZ domain of CASPR2 also interacts with PAR3, a cell polarity protein that regulates several aspects of brain development, including apical–basal polarity, asymmetric division of neural progenitors, migration, and process outgrowth, as well as the endocytosis and cellular localization of CASPR2 [162]. It is interesting that CASK and PAR3 are both critical for neurodevelopment, and that they bind to the same PDZ region of CASPR2, but it remains unclear whether these interactions impact one another or what downstream effects, if any, this may have on development [162].

Exogenous or overexpression of CICD is able to rescue some of the social and behavioral deficits in *Cntnap2* KO mice, and similar results have been shown with administration of oxytocin or mTOR pathway inhibitors [156,161]. Oxytocin has been shown to have a critical window during neonatal development where it plays a role in neuroinflammation and brain structure, including promoting cell survival, supporting neurite outgrowth and circuit formation, and facilitating the excitatory/inhibitory switch of GABA [163]. Thus, rescue of these social and behavioral deficits in CASPR2-deficient mice is most effective with chronic administration during that early postnatal period [147]. Additionally, the mTOR pathway is a critical regulator of neurodevelopment and overactivation of this pathway causes epilepsy and NDD, including tuberous sclerosis and focal cortical dysplasia—though the link, if any, between the mTOR pathway and variants in *CNTNAP2,* is unknown.

Loss of *CNTNAP2* has been shown to cause several cellular and structural brain abnormalities. For example, histology of human CDFE resections showed cortical thickening with an increased density of neurons and astrocytes, ectopic neurons in subcortical white matter along with overall blurred margin between white and gray matter, presence of large or binucleated neurons, abnormal dendritic morphology and orientation, as well as the presence of neurons that were abnormally organized into packed clusters or radial columns [145]. However, these malformations were found to be more diffuse across the tissue than what has typically been seen in other focal cortical dysplasias such as tuberous sclerosis [145]. Furthermore, *Cntnap2* KO mouse models recapitulate many of the structural and behavioral phenotypes seen in humans [146,149,156,158,164,165]. These mice had impaired neuronal migration, ectopic localization of later-born Cux1+ neurons in deep layers of the cortex, and a reduced number of cortical interneurons [146].

### 3.4. DENND5A

DENN domain-containing 5A (*DENND5A*) is associated with DEE characterized by seizures, abnormal brain development, DD, severe ID, lack of speech, and hypotonia [166]. Seizure onset typically occurs within the first 5 months of life and is generally medically refractory [166]. Neuroanatomical abnormalities are heterogenous, but include microcephaly, ventriculomegaly, pachygyria, and white matter hypoplasia [166].

DENND5A, also known as Rab6-interacting protein 1 (R6IP1), was initially characterized as a RAB-activated guanine nucleotide exchange factor (GEF) that interacts with Rab6 and Rab11 to regulate membrane trafficking [166,167,168]. In addition to membrane trafficking through regulating Golgi and recycling endosomes, Rab6 and Rab11 also play a role in mitotic regulation [166,167]. Depletion of Rab6 prevents cells from exiting mitosis via Mad2-dependent metaphase arrest, despite normal chromosomal alignment, which leads to cell death [169]. Rab11, on the other hand, is necessary for cytokinesis and facilitates cleavage furrow development and cell abscission [167]. Depletion of DENND5A blocks the metaphase/anaphase transition through activation of the Mad2 spindle checkpoint, which suggests that DENND5A acts as an effector of Rab6 during mitosis. Additionally, *DENND5A*-deficient cells that did bypass metaphase were binucleated, possibly due to the failure of Rab11-dependent cytokinesis [167]. Therefore, DENND5A supports mitosis through regulating both Rab6 and Rab11.

Along with supporting cell cycle progression during development, DENND5A is necessary to maintain potency of stem cells and neural progenitors. In addition to Rab6 and Rab11, DENND5A also interacts with PALS1 and MUPP1, two components of the Crumbs apical polarity complex [166]. This interaction is necessary for proper alignment of mitotic spindles and orientation of apical neural progenitors in symmetric cell division [166]. For example, loss of *DENND5A* inhibits symmetric cell division and enhances neuronal differentiation of hiPSCs [166].

Not only does DENND5A regulate development by enhancing stemness, it also does so by inhibiting differentiation signaling. Loss of *DENND5A* in neural progenitor cells induces neuronal differentiation and the formation of β-III tubulin-positive processes [166]. *Dennd5a* KO mice have a reduction in neural stem cells, indicating a depletion of the progenitor pool, as well as premature neuronal differentiation as indicated by significant increase in post-mitotic neurons [166]. During differentiation, *DENND5A* KO cells treated with NGF develop significantly longer neurites and have an increased surface area [168]. Cells also had an increased expression of neurotrophic receptors *TrkA* and *TrkB*, along with elevated Erk activation [168]. Furthermore, this Erk overactivation was observed even in the absence of NGF [168]. Interestingly, similar phenotypes of brain malformations related to impaired symmetric division of neural progenitors and premature differentiation were also seen in both mice and zebrafish [166,170].

Overall, DENND5A is a crucial regulator of neuronal progenitor proliferation, acting both as a pro-mitotic factor, as well as an inhibitor of differentiation. As a result, loss of *DENND5A* induces cell cycle exit and causes daughter cells to prematurely enter a fate-committed state during development, ultimately shortening the period of neurogenesis. This impairment of neuronal progenitor proliferation could explain the abnormal brain development that has been seen in patients, such as microcephaly and pachygyria, and lead to impaired connectivity that could explain ID and seizure phenotypes.

### 3.5. ALDH7A1

Aldehyde dehydrogenase 7 family member A1 (*ALDH7A1*) causes pyridoxine (Vitamin B6)-dependent epilepsy which is characterized by neonatal-onset seizures that are not responsive to standard ASMs but are typically well controlled by pyridoxine supplementation [171,172,173,174,175]. However, significant intellectual disability (ID) and developmental delay (DD) are still observed in at least 75% of individuals despite seizure control in response to pyridoxine [172,175,176]. While some evidence suggests that earlier intervention with pyridoxine treatment alone may help to lessen the severity of neurodevelopmental (NDD) outcomes [172,176,177], triple therapy of lysine restriction and arginine supplementation in addition to pyridoxine treatment has shown greater success in improving neurodevelopmental and cognitive outcomes [172,174,177,178].

*ALDH7A1* encodes alpha-aminoadipic semialdehyde dehydrogenase or antiquitin-1, which is a critical enzyme for lysine catabolism [172,174]. This process modulates cellular energy consumption to maintain homeostasis under hypoxic and starvation conditions [179]. Deficiency of antiquitin-1 results in the accumulation of the lysine intermediates α-amino adipic semialdehyde (α-AASA) and Δ-1-piperideine-6 carboxylate (Δ1-P6C), which can interact with and inactivate Vitamin B6, thereby leading to pyridoxine-dependent seizures [172,174,180]. This suggests that there are two separate pathogenic mechanisms that result from antiquitin-1 depletion, which require distinct therapeutic approaches, the first being epileptogenic sequestration of Vitamin B6, and the other being dysregulated lysine metabolism associated with ID/DD. Considering that the modest improvement to ID/DD symptoms by pyridoxine monotherapy is only seen with early intervention, that the addition of lysine restriction and arginine treatment for a triple therapy is more effective at decreasing ID/DD severity but is still limited, and that symptom onset is typically seen at neonatal stages, it is suggested that antiquitin-1 deficiency impacts prenatal brain development. Interestingly, there are several other genes (*TNSALP*, *PIGV*, *PIGL*, *PIGO*, *PNPO*, *PROSC*, *MOCS2*, or *ALDH4A1*) that have also been linked to pyridoxine-responsive epilepsies accompanied by ID/DD that is not prevented by pyridoxine treatment, despite obtaining epilepsy control [175]. This further supports the possibility that developmental etiology of epilepsy and neurodevelopmental disorders are distinct for this set of genes. In fact, ALDH7A1 seems to play a complex role in proliferation, migration, and differentiation of neurons during development. Antiquitin-1 has been shown to support NADH-dependent ferroptosis suppressor protein 1 (FSP1) activity and protect against ferroptosis-mediated cell death [180]. In mice, ALDH7A1 deficiency impairs pyrimidine biosynthesis, hindering normal proliferation and differentiation of neural stem cells (NSCs), even accompanied by pyridoxine treatment [181]. Interestingly, *ALDH7A1* expression is increased during G(1)-S phase transition of cell cycle progression, leading to accumulation of antiquitin-1 in the nucleus, while shRNA KD leads to altered expression of cell cycle regulators [182]. In postmortem specimens, cortical cytoarchitecture similar to Type Ia focal cortical dysplasia (FCD) was observed [183]. Furthermore, loss of *ALDH7A1* in mice caused dysregulation of astrocyte-derived matrix Gla protein (MGP) and led to impaired dendritic spine development, independent of seizures [184]. Both dendritic arborization and cognitive phenotypes were rescued by MGP activation [184].

### 3.6. RELN

Reelin (*RELN)* is a large extracellular matrix glycoprotein that was first discovered as the gene responsible for the *reeler* mouse phenotype and has since been established as a critical neurodevelopmental gene. Variants in *RELN* can cause epilepsy, ASD, and brain malformations—particularly lissencephaly with cerebellar hypoplasia [8,185,186,187,188,189,190,191,192,193]. Additionally, it is also associated with schizophrenia, bipolar disorder, depression, ID, and Alzheimer’s disease [186,191,194,195].

During cortical development, reelin is predominately expressed and secreted by the transient Cajal–Retzius cells within the marginal zone and regulates the migration of post-mitotic neurons as they travel from the ventricular zone, through the cortical plate, along radial glial fibers [186,194,196,197,198]. Reelin-dependent regulation of neuronal migration is critical for the laminar development of the cortex, whereby sequential rounds of newborn neurons will travel along the fibers of radial glial cells that extend out towards the pial surface, migrating past earlier-born neurons before detaching. As a result, the cortex is organized in a temporal-dependent “inside-out” layering pattern [199]. The characteristic cytoarchitectural phenotype associated with impaired reelin signaling is an inverted, “outside-in,” organization of the cortex [186,191,198,199].

Neurons sense reelin levels through the receptors—very low-density lipoprotein receptor (VLDLR), apolipoprotein E receptor 2 (ApoER2), and α3β1 integrin—in order to induce tyrosine phosphorylation and subsequent degradation of the cytoplasmic adaptor protein, disabled-1 (Dab1) [200,201]. Receptor binding also results in endocytosis and proteolytic cleavage of reelin, which, along with the secretion of proteases that act on reelin in the extracellular matrix, results in functional fragments that play a complex role in fine-tuning the intensity and timing of reelin signaling [202,203,204]. Dab-1-dependent reelin signaling can modulate several downstream signaling pathways, including PI3K/Akt, Lis1, Crks/C3G, MAPK, and Erk1/2, although this may vary by cell type [205,206]. Reelin signaling also regulates Golgi translocation and remodeling for proper orientation and leading-edge dynamics during migration [207]. Additionally, reelin also regulates the ratio and quantity of GABAergic neuronal subtypes within the hippocampus, cell cycle exit, and differentiation of neural precursors, and decreases the number of neuronal and glial cells [195,206].

Impaired reelin signaling can also alter neuronal morphology, including diminished dendritic complexity [208,209]. Chronic supplementation of reelin in vitro has been shown to rescue these morphological changes, and a singular in vivo injection of recombinant reelin into the lateral ventricles rapidly induces Dab1 degradation and causes long-lasting impact on dendritic morphology, synaptic plasticity, and learning [200,209]- a clear opportunity for therapeutic advances going forward.

### 3.7. PCDH19

Protocadherin 19 (*PCDH19*) is a cause of early infantile epileptic encephalopathy as well as neurodevelopmental disorders including ASD, ID, ADHD, developmental delay, and psychiatric features [171,210,211]. As an X-linked gene, phenotypes present in a sex-specific dimorphic pattern, whereby heterozygous females, but not hemizygous males, are affected [210,211,212,213]. Affected males with postzygotic somatic variants have been reported, but typically hemizygous men have ASD but not epilepsy [211,212,213]. In affected individuals, fever-sensitive seizures begin during infancy, but often enter remission in adolescence. Some individuals have symptoms that resemble Dravet syndrome, a severe early-onset childhood epilepsy that is highly medically refractory, but that is associated with variants in the sodium channel-encoding gene *SCN1A* [212,214]. ASM resistance in *PCDH19*-related seizures is highest during early childhood and tends to progressively improve with age. Despite this, early intervention is beneficial to reduce the overall seizure load and is also linked to better long-term cognitive outcomes [215].

PCDH19 is a cell–cell adhesion transmembrane protein in the cadherin family [211,212,216]. During early embryonic development, *PCDH19* is expressed in pluripotent cells and localizes to one pole, regulating cell polarity and defining apico-basal organization during neural tube development [216]. PCDH19 is also important for the regulation of symmetric vs. asymmetric division of progenitor cells by localizing into two poles during initial stages of proliferation, modulating mitotic spindle organization and cleavage plane orientation [216].

In addition to proliferation, PCDH19 also plays a role in neuronal migration and morphology [211,214,216]. The cytoplasmic domain of PCDH19 can regulate cytoskeletal remodeling through modulating the WAVE regulatory complex, thereby controlling both cell migration and axonal outgrowth [211]. In vivo, heterozygous *PCDH19*, but not homozygous KOs, caused impaired migration and survival-developing interneurons in a non-cell-autonomous manner [211].

### 3.8. CDKL5

Cyclin-dependent kinase-like 5 (CDKL5) is an X-linked serine/threonine protein kinase (previously known as STK9); variants in *CDKL5* cause genetic epilepsy and it is also associated with syndromic ASD, severe DEE, and clinical features similar to Rett syndrome and Angelman syndrome [171,217,218,219,220,221]. They are also associated with early-onset refractory seizures, DD, ADHD, ID, impaired vision-based features, as well as sleep disturbances and gastrointestinal issues [171,217,218,220,221].

Phenotypic severity is similar between males and females, but overall females are more commonly affected, and variability in X-chromosome inactivation patterning and postzygotic mosaicism also likely contributes to variable phenotypic severity [218]. However, brain abnormalities such as atrophy and white matter hyperintensities are more commonly seen in males, although these findings are overall non-specific compared to other DEEs [217]. Therapeutic approaches including ASMs, ketogenic diet, and vagal nerve stimulation have limited success in gaining long-term seizure control [217,222]. However, a recent study was able to rescue some pathological and behavioral phenotypes in *Cdkl5* KO mice via intracerebroventricular-injected AAV-based gene delivery. Unfortunately, translational potential may be limited due to the broad distribution and high AAV doses required [223].

*CDKL5* is predominately expressed in glutamatergic and GABAergic neurons and regulates neuronal differentiation via morphological and synaptic maturation of neurons during both development and adulthood [217,220,223,224,225]. During neuronal maturation and synapse formation, CDKL5 localization is prevalent in neuronal growth cones [220]. It also plays a role in synapses by regulating Bdnf/TrkB signaling of adult glutamatergic neurons and by regulating phosphorylation of voltage-gated calcium channel CACNA1E [219,226].

Phosphorylation targets of CDKL5 are involved in a range of cellular processes that are critical for brain development, and in turn, many of them are also associated with epilepsy and/or NDDs [220,227,228]. For example, ARHGEF2, DLG5, SHTN1, IQGAP1, EB2, MAP1S, CEP131, and AMPH1 are all involved in cytoskeletal regulation, including cell polarity and symmetric division of proliferating neural progenitors, cytoskeletal dynamics during neuronal migration including cilia formation and focal adhesions, and axonal outgrowth and vesicle trafficking during neuronal differentiation and maturation [220,227,228]. Additionally, several CDKL5 substrates, such as ELOA, EP400, TTDN1, MECP2, DNM1, HDAC4, SMAD3, and SOX9, regulate nuclear functions, including transcriptional regulation, chromatin structure, and DNA repair [220,227,228]. CDKL5-mediated SMAD3 activity has been shown to protect neurons from neurotoxic- or excitotoxic-induced cell death [221].

### 3.9. TSC1/TSC2

Loss-of-function (LoF) variants in either tuberous sclerosis complex 1 or 2 (*TSC1* OR *TSC2,* respectively) cause an autosomal dominant disorder (tuberous sclerosis complex; TSC), characterized by non-cancerous tumors that can occur in multiple organs, including brain, eyes, heart, kidneys, lungs, and skin [229,230,231,232,233,234,235,236]. Clinical manifestations of TSC are highly variable based on tumor localizations, with neurological disorders associated with brain lesions being especially prominent [232,237]. Some of the most common phenotypes of TSC are seizure disorders and TSC-associated neuropsychiatric disorders (TANDs), including epilepsy, infantile spasms, DEE, ASD, ADHD, and ID [8,230,231,232,235,237,238,239]. Epilepsy is one of the most common phenotypes of TSC, occurring in approximately 80% of cases, and is often associated with early onset, medically refractory seizures, and multifocal EEG abnormalities that are thought to originate from TSC-related focal lesions within the brain [229,232,237]. Brain lesions in TSC, such as focal cortical dysplasia (cortical tubers), subependymal nodules (SENs), and subependymal giant cell astrocytomas (SEGAs), are caused by localized disruptions to cortical cytoarchitecture and result in structural abnormalities that are generally detectable by MRI and can be potential targets for surgical resection [230,231,232,240].

Histopathological analysis has shown that cortical tubers exhibit both organizational and cellular abnormalities. Altered cortical cytoarchitecture can range from disorganized lamination to a complete loss of cortical layering [232,237]. Aberrant cell morphologies include dysmorphic neurons with abnormal somatic, axonal, and dendritic architecture, as well as astrogliosis and giant cells [229,231,232,237,241]. Cortical tubers form during fetal brain development and have been detected by neuroimaging as early as 20 weeks gestation [232,237]. In addition to organizational and morphological changes, markers of enhanced mTOR pathway activation are also present in TSC tuber samples, a hallmark feature [237,242,243,244].

Mechanistically, cortical tuber formation and the cytoarchitectural abnormalities therein result from mTOR pathway hyperactivation-induced changes in neuron migration, polarization, maturation, and morphology. Indeed, several experimental models have shown that loss of *Tsc1/2* results in decreased reelin signaling (described above; [245]) and the processes that regulate neuronal polarity [246]. Further, giant cells within cortical tubers retain an immature phenotype and do not possess the hallmark electrophysiological properties of mature neurons [247]. Together, these changes (and others) are hypothesized to set up abnormal networks within the cortex that drive epilepsy in TSC. Interestingly, mTOR pathway hyperactivation within the cerebellum is hypothesized to drive ASD phenotypes in TSC. Cerebellar Purkinje cells in Tsc1 mutant mice—where ASD-like behaviors are observed—have abnormal morphology, decreased excitability, and are fewer in number than controls [248,249]. Further, loss of *Tsc1* in striatal dopaminergic neurons results in cognitive abnormalities similar to those observed in individuals living with TAND [250]. While the exact mechanisms by which abnormal cortical, striatal, and cerebellar function produce ASD, epilepsy, and TAND in TSC is unknown, it is clear that the same genetic variant can have cell type-specific consequences that drive different disease phenotypes.

### 3.10. TANC2

*TANC2*-related disorder (TRD) is a newly identified but poorly understood condition that affects multiple organ systems, but most predominately affects brain development. It is caused by autosomal dominant LoF variants within *TANC2*, resulting in haploinsufficiency [251,252,253,254,255,256,257,258]. While the clinical manifestations of TRD are highly variable, common phenotypes include ID, DD, ASD, epilepsy, microcephaly, craniofacial dysmorphic features, hypotonia, as well as neuropsychiatric disorders such as schizophrenia [254,255,256,257,258,259,260,261,262,263]. In cases associated with epilepsy, seizure types and severity are highly variable as well, and several cases of refractory seizures and epileptic encephalopathy have been reported [254,256,258]. In addition to TRD, genome-wide association studies (GWASs) and transcriptomic analysis revealed that *TANC2* is associated with cardiovascular disease, multiple sclerosis, Alzheimer’s disease, and several types of cancers [264,265,266,267,268,269].

Tetratricopeptide repeat, ankyrin repeat, and coiled-coil containing 2 (TANC2) and its paralog, TANC1, are named based on putative structural motifs commonly involved in protein–protein interactions, multiprotein complex mediation, and molecular spacing [125,251,270,271,272,273]. They were first discovered as mammalian homologs to Rols/Ants in *Drosophila*, a scaffolding protein that interacts with intracellular structural proteins, including D-titin and alpha-actinin, as well as the cell adhesion molecule, dumbfounded (Duf), to facilitate myoblast fusion during muscle development [251,273,274,275,276,277,278]. The first characterization of the putative mouse orthologs of Rols/Ants, mants1 (Tanc1) and mants2 (Tanc2), showed differential expression patterns during embryonic development [278]. At E11.5, Tanc1 showed broad, transient expression across embryonic mesodermal mouse tissue, consistent with expression patterns of Rols/Ants during *Drosophila* muscle development [278]. On the other hand, strong expression of Tanc2, but not Tanc1, were detected at this time point in neural mouse embryonic tissues, including neural tube and dorsal root ganglia [278]. Subsequent studies found both Tanc1 and Tanc2 to be expressed in the brain, albeit with different spatiotemporal patterning, with enriched localization within neuronal synapses [251,273].

Despite the many differences between muscle and neuronal development, the fusion structure formed between plasma membranes of founder cells and fusion-competent myoblasts, called a fusogenic synapse, has been compared to both neuronal and immunological synapses due to analogous processes, including cytoskeletal modulation, cell adhesion, and vesicle trafficking [278]. For example, the cell adhesion molecules Kirrel1/3 are mammalian homologs of Duf that are also expressed in the developing and adult brain; they interact with the synaptic scaffolding protein CASK and are believed to play a role in neurodevelopment and synaptogenesis [279,280,281,282]. Kirrel1 facilitates Hippo pathway-mediated cell–cell contact inhibition by acting as a negative feedback regulator of YAP activity [283]. Interestingly, TANC2 also interacts with scaffolding proteins in the postsynaptic density, such as PSD-95, and may also interact with proteins involved with regulating the Hippo pathway, including LATS2, PPP1CA, PPP1CC, YWHAB, and ZYX [251,252,273]. Furthermore, Kirrel3 supports cell recognition mediated synaptogenesis and colocalizes with Golgi apparatus and synaptic vesicles; also, it is associated with NDDs, including ID, ASD, cognitive and psychomotor delays, cerebellar hypoplasia, dysmorphic features, and Jacobsen syndrome [281,284,285,286,287,288]. Whether Tanc1/2 and Kirrel1/3 have overlapping mechanisms in neurodevelopment is unclear, but Kirrel3 has been detected as a Tanc2 interactor in the adult mouse brain via high-throughput co-fractionation mass spectrometry [289].

Myo18a is another example of a Tanc1/2-interacting protein that is involved in both brain and muscle development, and whose Drosophila ortholog, Mhcl, interacts with Rols/Ants during myoblast fusion [290]. This unconventional myosin has a multifaceted role in intracellular trafficking and cytoskeletal regulation, and is also associated with NDDs, including early infantile epileptic encephalopathy [291]. Expression of *Myo18a* supports cellular protrusions and focal adhesions turnover and is essential for actin retrograde treadmilling, actomyosin assembly, and force generation that drives cell motility [290,292,293,294,295,296]. Additionally, colocalization of Myo18a in dendritic spines and *trans* Golgi promotes actin assembly associated with synapse maturation and the formation of vesicles that are trafficked to the plasma membrane, respectively [290,293,297]. Interestingly, Tanc2 also plays a role in synaptic vesicle transport. Within dendritic spines, TANC2 is involved in capturing dense core vesicles that are shuttled by the kinesin KIF1A along dendritic microtubules in response to calcium-dependent calmodulin activity [298]. Similarly to Kirrel1/3, whether there is a relationship between Myo18a and Tanc2 in the context of neurodevelopment and vesicle transport remains to be elucidated. However, given the centrality of Tanc2 in modulating dendritic structure at synapses, TRD may represent a keystone disorder where further research will reveal convergent mechanisms that impact our understanding of other disorders.

## 4. Discussion

Overlapping features and a high rate of co-occurrence across NDDs makes differential diagnosis challenging, especially when the predominant diagnostic classifications of these disorders rely heavily on observational behavioral phenotypes [1,2,3,4,5,17] (Table 1; Supplementary Materials Table S1). This is highlighted in the evolution of both terminology and official diagnostic classification of ASD in the DSM—particularly a shift from discrete categories to instead take a dimensional approach of considering autistic traits to lie across a spectrum [2,299]. However, it is important to note that a broader dimensional approach to classifying and diagnosing ASD still relying heavily on behavioral-focused approaches is susceptible to confounding influence of social stigma, gender, socio-economic status, and cultural standards in how diagnostic criteria are clinically assessed. This can lead to both oversimplification of phenotypic heterogeneity and underrepresentation of affected individuals within certain demographics [299,300,301]. Furthermore, when comorbidity is common and the boundaries between NDDs are unclear, diagnostic overshadowing can prevent or delay the identification and treatment of some phenotypes [2,4,8]. These diagnostic complexities and heterogeneity of phenotypic presentation of neurodevelopmental disorders impose significant challenges in the use of forward genetics or a phenotype-to-genotype approach to elucidating pathogenic mechanisms [2,301]. Thus, the goal of gaining deep mechanistic insights into these NDDs will allow for the development of target therapies, together with greater clinical clarity and societal understanding of NDDs broadly.

Over the last several decades, significant technological advancements in whole-genome sequencing, neuroimaging resolution, molecular genetics, and experimental modeling tools, paired with big-data computational analysis, have significantly improved our understanding of NDD and is helping to inform and refine phenotypic descriptions. These approaches have uncovered several risk genes and molecular pathways that are linked to one or more complex CNS disorders, including epilepsy, NDDs, and neuropsychiatric disorders, leading to mechanistically informed targeted therapies [2,302,303]. However, it has become increasingly clear that these disorders are not just phenotypically heterogeneous, but genetically and molecularly complex as well. Distinguishing between convergent and divergent pathogenic mechanisms of these disorders, and subsequently identifying therapeutic targets, are impacted by several factors, including variable penetrance, functional outcomes of distinct pathogenic variants within the same gene, spatiotemporal regulation of gene expression, cell autonomous and non-cell autonomous effects, and somatic and germline variant types—all of which will require further work to be understood fully.

While establishing meaningful genotype–phenotype relationships has proven challenging, mechanisms involved with synaptic structure, connectivity, and plasticity are commonly associated with pathogenesis of epilepsy and ASD. This is supported by the fact that many antiseizure medications (ASMs), which were designed to primarily treat epilepsy as an independent etiological disorder and which typically modulate neurotransmission or ion channel activity as their dominant mode of action, can in some cases provide cognitive, behavioral, or psychological benefits [8,304]. Unfortunately, cases of refractory epilepsy persist at significant rates. Even within the context of pathogenic variants in ion channels, outcomes vary widely, but commonly, early therapeutic intervention can significantly improve seizure and developmental outcomes.

**Figure 1:**
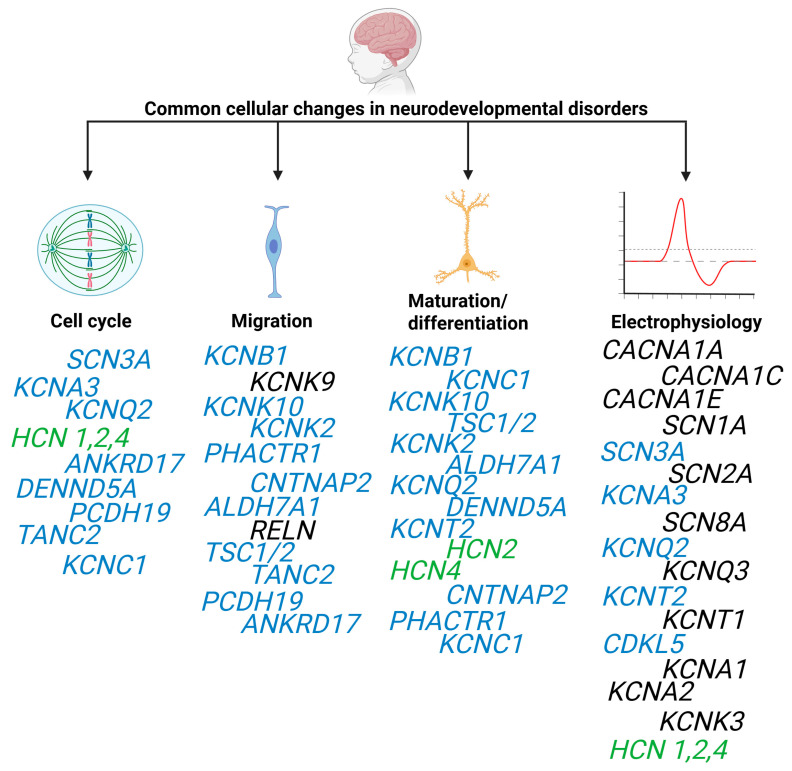
Summary of cellular changes associated with NDD genes. Under each cellular change category, gene variants associated with on-process change are in black, two changes in biological processes are in blue, and three changes are in green. Cell cycle = changes in processes related to cell division. Migration = changes associated with neuronal migration during brain development. Maturation/differentiation = changes that result in abnormal development of neurons themselves. Electrophysiology = changes associated with action potential firing at the single cell or network level. This list includes only those detailed in this manuscript. Created in BioRender. Iffland, P. (2026) https://BioRender.com/07nqn9s (accessed on 22 December 2025).

Many pathogenic genes linked between epilepsy and NDDs, including ion channels, are expressed during prenatal and neonatal development and are functionally and temporally associated with stages of brain development that proceed the establishment of mature network connections, indicating that early developmental impairments can have a cascading effect on downstream neuronal activity (Figure 1). This is particularly apparent when considering the pathogenic genes and molecular mechanisms that cause malformations of cortical development (MCDs). The identification of common mechanistic pathways during embryonic development that cause malformations of cortical development (MCDs) and which are highly associated with epilepsy, autism, and other NDDs, has led to new therapeutic approaches.

The brain is an extraordinarily complex system and is relatively modern in evolutionary terms, especially to the level of complexity present in humans. Proper development requires a highly specialized sequence of events. This is particularly true for neurons as compared to other cell types. Once differentiated and matured, neurons are predominately post-mitotic, which means early developmental regulation of cell cycle progression and cell death are essential for regulating the correct quantity of neurons. Because these biological processes occur during an early developmental window, pathogenic variants that impair their regulation can impact downstream neurological functions and cause particularly severe presentations of epilepsy, ASD, and NDDs. In those cases, high prevalence of refractory epilepsy indicates that treating seizures may only be addressing a symptom but not the root cause of the disease.

Similarly, the organization and cytoarchitecture of the brain relies on precise regulation of cell migration and process outgrowth. Furthermore, throughout development, cells progressively undergo multiple processes of cellular differentiation, from early stem cell states to neural progenitors, and eventually differentiated neurons and glial cells. Dysregulation of these processes can significantly affect the overall landscape of the brain, including populations of specialized cells, how those cells are organized, and ultimately how they mature, form synapses, and strengthen network connections. These neurodevelopmental processes do not occur as discrete temporal phases, as demonstrated in this review, and many of the genes and pathways that regulate these processes are interconnected. Thus, identifying these convergent processes in one disorder may facilitate understanding the pathogenesis of similar disorders.

The relationship between epilepsy and ASD is highly variable, depending on the gene and its functions. In some cases, they are unrelated and non-overlapping. However, for genes that are critical for early stages of brain development, there tend to be higher rates of comorbidity. However, even in that context, some genes seem to impact separate downstream processes that serve as distinct etiologies for epilepsy and NDDs, as indicated by the genes where therapeutics improve one but not the other. Alternatively, there may be a causative relationship (e.g., cognitive impairments or developmental regression caused by neurotoxicity of seizure activity). However, many of the molecular and cellular mechanisms behind brain development, epilepsy, ASD, and NDDs still remain unclear. Indeed, there is still much that remains unknown about the molecular function of most of these genes and the sequence of events that connect molecular function to network function to disease phenotype. Further, things like gene dose sensitivity, genetic imprinting, variant-specific changes in protein product and domain functionality, and other factors that impact variable expressivity and penetrance make it challenging to interpret the relationship between gene function and phenotype.

Many genes, especially ones that regulate broad cellular functions, such as proliferation, migration, and differentiation, are also likely to have syndromic presentations. Some of them are also linked to cancer development and progression, which further supports the importance of these genes in functional mechanisms of molecular and cellular biology. However, understanding these cellular mechanisms, especially when considering distinct functional outcomes across pathogenic variants, will be invaluable for identifying therapeutic targets and developing new treatment options.

There are several limitations that hinder the ability of investigators to clearly define disease mechanisms and implement novel therapies in NDDs. First, due to the rarity of some of these disorders, it is difficult to establish genotype–phenotype correlations, and the paucity of available samples makes it challenging to elucidate disease mechanism both within, and especially across, variants. Additionally, while the increased accessibility to whole genome sequencing has significantly expanded the number of identified pathogenic variants associated with NDDs, detection of somatic mosaic variants remains challenging in brain-specific disorders. GoF and LoF variants in the same genes can result in similar phenotypes, making understanding disease mechanisms even more challenging and can make large-scale clinical trials of novel therapies difficult. Additionally, many of these disorders arise in utero, making them difficult to treat from both a logistical and ethical standpoint. Lastly, current laboratory models of NDDs, particularly rodent models, fail to recapitulate many of the developmental processes that are altered in these disorders (see sections on *SNC3A* and *TSC).* While stem cell-based models have addressed some of these differences, they do not address all of them and can be technically complicated and expensive to perform on a large scale across genotypes.

## 5. Conclusions

Comorbidity of epilepsy with neurodevelopmental disorders (NDDs), particularly DD, ID, and ASD, are common. This is particularly true when the age of seizure onset is very young, during neonatal and infantile stages [305]. Furthermore, early age of onset in epilepsy is also commonly associated with symptom severity and treatment outcomes, including medically refractory seizures, status epilepticus, DEE, and SUDEP. Considerable progress has been made thus far in identifying genotype–phenotype relationships, coinciding with advancements in our understanding of molecular genetics and developmental biology. Attempts at finding targetable convergent mechanisms have hit several roadblocks, pointing to a much more complex etiology underlying these disorders. Across several identified risk genes, it is becoming clearer that different genetic variants can have drastically different functional outcomes on brain development and significantly different responses to treatment despite seemingly convergent mechanisms. Indeed, it is likely that some convergent mechanism will yield tractable therapeutic approaches across disorders. However, many NDDs may require specific therapy or a cocktail of therapies that work only for that disorder. To address these gaps in knowledge and clinical practice, precision-medicine approaches to target the gene variants themselves will be required or, alternatively, the implementation of new therapies that target critical molecular nodes downstream of the gene will be required. However, deeper mechanistic insights into disease pathogenesis will be necessary for many of these disorders before meaningful genotype–phenotype correlations can be made and tractible therapeutic interventions can be trialed.

## Figures and Tables

**Table 1:** Summary table of NDD gene variants with associated NDD name/category and CNS specific features. This table only includes NDD-associated genes that are the focus of analysis in this article, directly related to epilepsy/ASD, and have systematic mechanism studies, covering key pathways of brain development. They do not represent all associated genes. An expanded version of this table can be found in the Supplementary Materials Table S1. ASD = autism spectrum disorder, ID = intellectual disability, DD = developmental delay, ADHD = attention-deficit hyperactivity disorder, TAND = tuberous sclerosis complex-associated neuropsychiatric disorder, DEE = developmental and epileptic encephalopathy, * = no specific syndrome name.

Gene	Named CNS Syndrome	DEE?	Features
*CACNA1A*	*	Yes	ASD, DD, ID, hypotonia, ataxia, epilepsy
*CACNA1C*	*		ASD, DD, hypotonia, seizures
*CACNA1E*	*	Yes	ASD, DD, macrocephaly, epilepsy
*SCN1A*	Dravet Syndrome	Yes	epilepsy, ASD, ID, cognative decline
*SCN2A*	SCN2A-related nonspecific severe intellectual disability	Yes	epilepsy, DD, ID, ASD, ADHD, microcephaly, hypotonia, white matter defects
*SCN3A*	SCN3A-related focal epilepsy	Yes	epilepsy, ID, polymicrogyria
*SCN8A*	*	Yes	epilepsy, DD, ID, hypotonia, ataxia
*KCNQ2*	KCNQ2-related epileptic encephalopathy, KCNQ2-related benign neonatal epilepsy	Yes	epilepsy, infantile spasms, ID, DD, ASD
*KCNQ3*	*		ID, DD, seizure
*KCNA1*	*		ataxia
*KCNA2*	KCNA2-related epileptic encephalopathy	Yes	DD, ID
*KCNT1*	KCNT1-related epilepsy	Yes	epilepsy, ID, psychiatric conditions
*KCNT2*	*	Yes	developmental defects, ID
*KCNB1*	*	Yes	epilepsy, DD, ASD
*KCNC1*	*		myoclonic epilepsy, IDD, DD
*KCNK3*	*		cortical heterotopia
*KCNK9*	Birk-Barel Syndrome		speech and motor delay, ID, behavoral abnormalities
*KCNK2*	*		cortical heterotopia
*KCNK10*	*		cortical heterotopia
*HCN1*	atypical Rett syndrome	Yes	ID, DD, microcephaly, movement dysfunction, epilepsy
*HCN2*	*	Yes	ID, DD, epilepsy
*HCN4*	*		epilepsy, cortical malformations
*ANKRD17*	Chopra-Ameil-Gordon Syndrome		DD, ID, speech delay, ASD, ADHD, epilepsy
*ALDH7A1*	*		ID, DD, epilepsy
*DENND5A*	*	Yes	epilepsy, DD, ID, speech defecits, hypotonia, brain malformations
*CDKL5*	*	Yes	ASD, DD, ADHD, ID, imparied vision, sleep disturbances
*CNTNAP2*	Pitt-hopkins like syndrome, CDFE		ID, DD, epilepsy, ASD, ADHD, cortical malformations
*PHACTR1*	West syndrome	Yes	infantile spasms, developmental regression, ID, ASD, lissencephaly
*RELN*	*		ASD, ID, schizophrenia, bipolar disorder, depression, Alzheimers disease, lissencephaly
*PCDH19*	*	Yes	ASD, ID, ADHD, DD, psychiatric features, epilepsy
*TANC2*	Tanc2-related disorder		ASD, epilepsy, ID, DD, microcephaly, schizophrenia
*TSC1/2*	Tuberous Sclerosis Complex		ASD, ID, epilepsy, TAND

## Data Availability

No new data were created or analyzed in this study.

## Supplementary Materials

The following supporting information can be downloaded at: https://www.mdpi.com/article/10.3390/brainsci16010054/s1, Table S1: expanded genotype/phenotype data from Table 1.
